# Cross-species cluster co-conservation: a new method for generating protein interaction networks

**DOI:** 10.1186/gb-2007-8-9-r185

**Published:** 2007-09-05

**Authors:** Anis Karimpour-Fard, Corrella S Detweiler, Kimberly D Erickson, Lawrence Hunter, Ryan T Gill

**Affiliations:** 1 Center for Computational Pharmacology, University of Colorado School of Medicine, Aurora, Colorado 80045, USA; 2 MCD-Biology, University of Colorado, Boulder, CO 80309, USA; 3 Department of Chemical and Biological Engineering, University of Colorado, Boulder, CO 80309, USA

## Abstract

Co-conservation (phylogenetic profiles) is a well-established method for predicting functional relationships between proteins. Several publicly available databases use this method and additional clustering strategies to develop networks of protein interactions (cluster co-conservation (CCC)). CCC has previously been limited to interactions within a single target species. We have extended CCC to develop protein interaction networks based on co-conservation between protein pairs across multiple species, cross-species cluster co-conservation.

## Abstract 2

Cluster Co-Conservation (CCC) has been extended to a method for developing protein interaction networks based on co-conservation between protein pairs across multiple species, Cross-Species Cluster Co-Conservation (CS-CCC).

## Background

The exponential increase in sequence information has widened the gap between the number of predicted and experimentally characterized proteins. At present, about 400 microbial genomes are fully sequenced. The prediction of protein function from sequence is a critical issue in genome annotation efforts. Currently, the best established method for function prediction is based on sequence similarity to proteins of known function. Unfortunately, homoogy-based prediction is of limited use due to the large number of homologous protein families with no known function for any member. An alternative method for predicting protein function is the phylogenetic profiles approach, also known as the co-conservation (CC) method first introduced by Pellegrini *et al*. [1]. Co-conservation predicts interactions between pairs of proteins by determining whether both proteins are consistently present or absent across diverse genomes [2-8]. CC methods have been shown to be more powerful than sequence similarity alone at predicting protein function.

Even though all CC methods rely on the premise that functionally related proteins are gained or lost together over the course of evolution, several different strategies for performing CC studies have been reported. For example, Date *et al*. [7] used real BLASTP best hit E-values normalized across 11 bins instead of binary classification for conservation, while Zheng and coworkers [9] constructed phylogenetic profiles using presence/absence of neighboring gene pairs. Alternatively, Pagel *et al*. [10] constructed phylogenetic profiles between domains, instead of genes, and then created domain interaction maps. Barker *et al*. [11] applied maximum likelihood statistical modeling for predicting functional gene linkages based on phylogenetic profiling. Their method detected independent instances of protein pair correlated gain or loss on phylogenetic trees, reducing the high rates of false positives observed in conventional across-species methods that do not explicitly incorporate a phylogeny [11].

**Figure 1:**
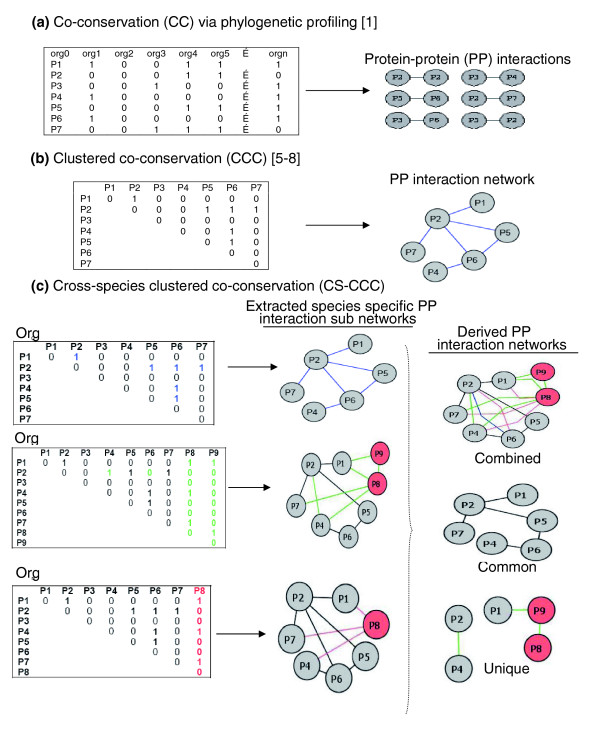
CS-CCC builds on information generated via previously described CCC methods by comparing conserved network interactions across multiple species. CCC methods start by mapping **(a) **co-conserved proteins pairs to **(b) **large protein interaction networks. **(c) **CS-CCC extends this approach by comparing proteins and associated links within such interaction networks to identify the combined set of network interactions as well those interactions that are unique to individual species or common across multiple species. Clusters from three organisms are shown, but the method could examine any genome versus any number of genomes (the unique differences between an organism of choice and each organism are shown in different colors while conserved proteins across species are shown in gray). Common network interactions are shown in blue while unique interactions are shown in either green or red. Org (organism); org0 (organism of choice); P (protein).

Currently, several web-based databases that compile predictions of protein-protein interactions are available, for example, PLEX [7], String [8], Prolinks [6], and Predictome [5]. These databases use various methods, including CC, to organize groups of proteins within individual species into clusters (cluster co-conservation (CCC)) that represent predicted protein interaction networks. Here, we have investigated the degree to which these within-species clusters are conserved across different species, using an automated method for comparing phylogenetic profiling based CCC across multiple species (CS-CCC; Figure 1). CS-CCC is essentially a meta-analysis of CCC that automates the identification of interactions that are uniquely present or absent across different species, which cannot be easily accomplished using existing methods. We have shown that this method increased groupings among proteins that function in distinct but coordinate processes and decreased groupings among proteins with unknown functions. This suggests that CS-CCC, in comparison to CCC, allows one to extend the network to better understand pathways involving proteins with multiple functions. Our intention for CS-CCC was that the identity of proteins present or absent in co-conserved clusters when evaluated across multiple species would facilitate the assignment of protein function, enable the development of novel and testable biological hypotheses, and provide experimentalists with the scientific justification required to test these hypotheses. We show these features through a number of different examples involving complex biological phenomena (that is, flagellum, chemotaxis, and biofilm proteins).

## Results

### Cross-species clustered co-conservation

**Figure 2:**
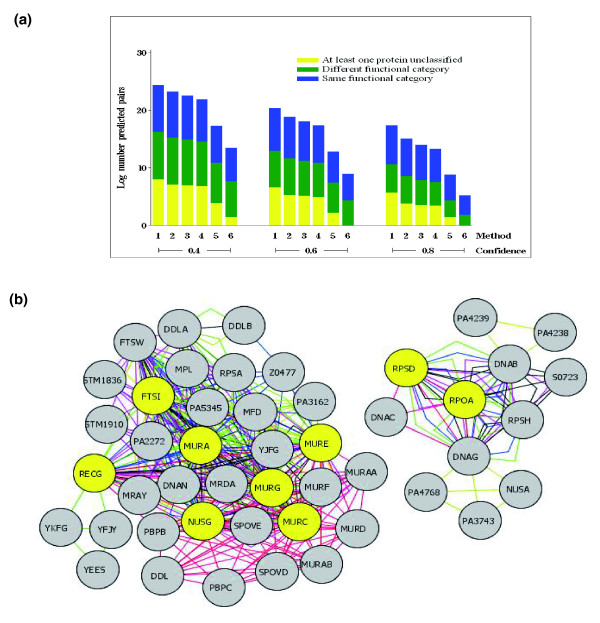
Assessment of CS-CCC Performance. **(a) **Comparison of COG functional categories of predicted pairs at three different confidence levels. The first method (1) used only *E. coli *K12. Each subsequent method added an additional (underlined) bacterial strain. 1, *E. coli *K12; 2, *E. coli *K12 and *E. coli *O157; 3, *E. coli *K12, *E. coli *O157 and *S. flexneri*; 4, *E. coli *K12, *E. coli *O157, *S. flexneri*, and *S. typhimurium *LT2; 5, *E. coli *K12, *E. coli *O157, *S. flexneri*, *S. typhimurium *LT2, and *P. aeruginosa*; 6, *E. coli *K12, *E. coli *O157, *S. flexneri*, *S. typhimurium *LT2, *P. aeruginosa*, and *B. subtilis*. The percentage of predicted interactions involving proteins from the same functional category (blue), different functional categories (green), or involving at least one protein that is unclassified (yellow) are depicted. **(b) **The CS-CCC network generated from the complete set of proteins included in the green bar of (a) for a confidence of 0.8, 6 species. A total of nine proteins (yellow nodes) and six-paired interactions were included in this group. The protein pairs and the classifications of each protein are as follows: (FtsI [M] and NusG [K]; MurE [M] and RecG [L]; MurG [M] and RecG [L]; MurC [M] and RecG [L]; MurA [M] and NusG [K]; RpoA [K] and RpsD [J]). M, cell envelope biogenesis, outer membrane; K, transcription; L, DNA replication, recombination and repair; J, translation, ribosomal structure and biogenesis. The edges are color coded for each species evaluated: *E. coli *K12, green; *E. coli *O157, blue; *Shigella flexneri*, black; *S. typhimurium *LT2, purple; *P. aeruginosa*, mustard; and *Bacillus subtilis*, red.

CS-CCC is based on the use of CC methods simultaneously across several species. As such, the reliability of the CS-CCC method is directly linked to the reliability of existing CC methods, which has been extensively documented [2-8]. Specifically, since CC methods produce protein-protein interactions involving proteins with previously uncharacterized functions, CC methods perform better than sequence similarity methods alone at predicting protein function. Here, we performed the same comparison to assess the performance of CS-CCC (up to six species) when compared to CCC alone (one species) (Figure 2a). The reliability of predicted protein interaction pairs was evaluated by using a combination of Clusters of Orthologous Groups (COG) functional categories, and The Institute for Genomic Research (TIGR) role categories (Additional data file 1). As the number of species included in our CS-CCC analysis increased, the number of predicted interactions involving proteins with unclassified functions decreased (yellow bars). Interestingly, at the lowest confidence level, the number of predicted interactions involving proteins from different functional categories increased with the number of included species. At the highest confidence level, grouping between proteins from the same functional category increased. For example, 56% of *Escherichia coli *K12 protein pairs (confidence level of 0.6) consisted of proteins within the same COG functional group, 19% of protein pairs were in different functional categories, and 25% had at least one unclassified member due to limited experimental data. As the number of species is expanded, these percentages range from 54-62%, 30-45%, and 0-10%, respectively. At the highest confidence level (0.8), the inclusion of 6 species resulted in almost 80% of the predicted interactions involving proteins from the same functional category. These results suggest that expanding the number of species included in the analysis, as provided for by CS-CCC, not only predicts interactions that are not predicted at different confidence levels used in CCC analysis, but also that the nature of such predicted interactions is fundamentally different. One explanation for such observations is that CS-CCC has improved capabilities for extending the protein interaction network to include the various functions required in complex biological processes (that is, regulatory relationships, nutrient transport/catabolism links, and so on). As an example of this possibility, in the CS-CCC analysis using all 6 bacterial species at confidence level 0.8 (the green bar on the far right on Figure 2a), there were 6 co-conserved protein pairs involving 9 total proteins that were not in the same COG functional category. When the larger network that these pairs fall into was extracted (Figure 2b), it became apparent that each of the proteins in question function within the context of two larger, coherent networks involving related processes. For example, *rpoA *and *rpsD *encode proteins of differing functions, yet their interaction is well conserved across multiple species within a 12-gene network of related functions. The remaining seven proteins of varying functions were also well conserved across multiple species in a larger network. These data suggest that the addition of multiple species to the analysis adds confidence to predicted interactions among proteins from different functional categories (that is, a meta-analysis). This point is exemplified via the color-coded, species specific arcs in Figure 2b, where it is clear that addition of multiple species both adds new interactions (that is, unique sub-networks) and reinforces the interactions predicted for comparison species.

### CS-CCC identifies interactions that could not be identified by CCC

**Figure 3:**
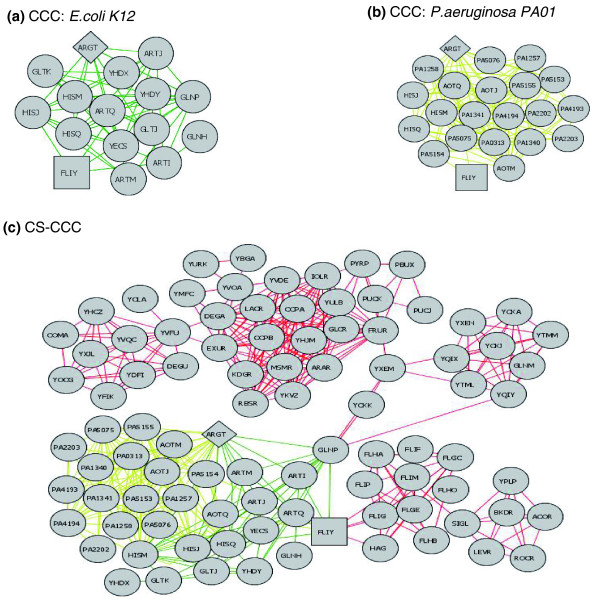
CS-CCC identifies protein interactions that could not be identified by CCC. **(a) ***E. coli *K12 cluster built around Arg*T*; **(b) ***P. aeruginosa *PA01 cluster built around ArgT; **(c) **an example of information revealed by CS-CCC but not by CCC. *E. coli *K12 proteins (green) that are co-conserved with *E. coli *ArgT (diamond) cluster were extracted. Then *P. aeruginosa *(mustard edge) and *B. subtilis *(red edge) proteins that are co-conserved with proteins in the *E. coli *ArgT cluster were extracted. Note that it is the *B. subtilis *network that shows a connection between amino acid biosynthesis proteins and flagellar proteins, via FliY (square). If only the *E. coli *cluster had been examined, as occurs using the CCC method, then this connection would have been missed.

Our analysis of CCC across six bacterial species indicated that CS-CCC revealed unique and useful information not provided by CCC alone. As one example, CS-CCC uniquely revealed that amino-acid biosynthesis and flagellar networks are connected via FliY (Figure 3c), a component of the flagella motor-switch complex that is predicted to transport amino acids [12]. Both *E. coli *and *Pseudomonas aeruginosa *ArgT networks revealed connections with the FliY protein (Figure 3a,b), but such networks did not include the extensive set of additional flagellar protein interactions predicted in the *Bacillus subtilis *network. Such information can be used to not only develop more precise hypotheses about protein function but also to provide the justification required to test such hypotheses. A second example of information uniquely revealed by CS-CCC suggests how the process of chemotaxis has evolved across species. A CS-CCC comparison of chemotaxis in *E. coli *K12 and *Salmonella *revealed that *Salmonella *lacks Tap, which transports maltose, but has Tcp, which transports citrate. In contrast, *E. coli *has Tap but lacks Tcp. CCC analysis alone does not capture this difference in chemotaxis responsiveness. As a final example, extending this CS-CCC analysis of chemotaxis proteins to include *P. aeruginosa *indicated new links among type IV pili and biofilm formation proteins [13,14], suggesting that the process of chemotaxis has evolved different functional relationships in different species. These three examples provide a simple demonstration of the ability of CS-CCC to predict unique and biologically informative interactions when compared to CCC alone. The next several sections elaborate upon the specific types of interactions that CS-CCC is uniquely suited at identifying.

### CS-CCC reveals how proteins that function in distinct but coordinated processes may have evolved

#### Chemotaxis

**Figure 4:**
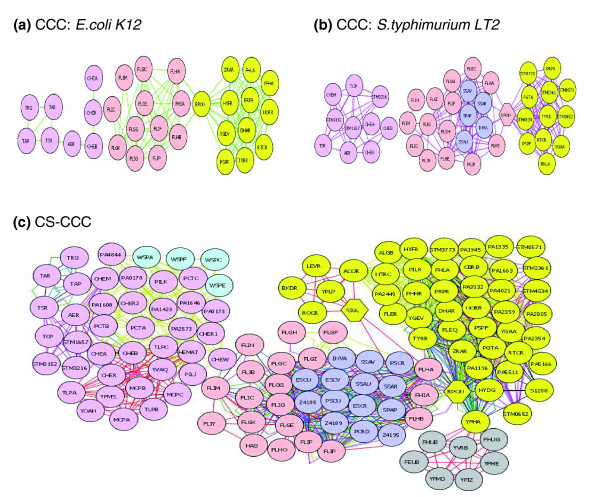
Co-conservation of chemotaxis and flagellar proteins. **(a) ***E. coli *K12; **(b) ***S. typhimurium *LT2; **(c) **across multiple species. Proteins are color coded base on function: chemotaxis, pink; biofilm, light blue; flagellar, light red; type III secretion, blue; and sigma factor and regulation, yellow. The gray proteins are *Bacillus *sigma factor and regulation that are co-conserved but were not identified by single species CC analysis. Edge color code: *E. coli *K12, green; *E. coli *O157, blue; *Shigella flexneri*, black; *S. typhimurium *LT2, purple; *P. aeruginosa*, mustard; and *Bacillus subtilis*, red.

Chemotaxis proteins are co-conserved across the examined bacteria (Figure 4). Three classes of proteins are essential for chemotaxis: transmembrane receptors, cytoplasmic signaling components, and enzymes for adaptive methylation. The transmembrane receptors are two-component signal transduction complexes called methyl-accepting chemotaxis proteins (MCPs). *E. coli *MCPs are Tsr, Tar, Trg, Tap, and Aer, and each recognizes specific sugars, amino acids or dipeptides (Figure 4a,c). Even though different bacteria have different MCPs, they are highly co-conserved among Gram-negative and positive bacteria. For example, *Salmonella *lacks Tap, which recognizes maltose, but has Tcp, a citrate sensor [15], which is co-conserved with the other *Salmonella *MCPs (Figure 4b,c). The cytoplasmic signaling components transmit signal between the MCP receptors and the flagellar apparatus. These proteins are CheA, CheW, CheY and CheZ, and they are not co-conserved among the bacteria. CheZ is not co-conserved because it has no homology across many bacteria [15]. CheY is likely not co-conserved because it functions with CheZ. CheA and CheW are sometimes co-conserved and sometimes not, which may suggest that they function independently in different bacteria. The enzymes for adaptive methylation, CheB and CheR, modulate signaling of the cytoplasmic proteins, and both of these proteins are highly co-conserved among all six bacteria. Thus, chemotaxis analysis illustrates two important points. First, the CS-CCC method reveals species differences in protein interaction, including co-conserved pairs that are unique to a given species or that are common across select species (Figure 4c). For instance, the sequences of CheA and CheW are conserved but the proteins are not co-conserved, suggesting that their interactions and functions may differ among bacterial species. Second, the CS-CCC method yields information that functional assays do not. For instance, different MCPs recognize different ligands and yet are co-conserved because they function in the same pathway.

#### Biofilm formation

Figure 4 shows a cluster containing proteins that function in distinct but inter-dependent processes. For instance, in *P. aerginosa*, flagella, chemotaxis machinery, and type IV pili are important for bacterial biofilm formation [13,14] and are co-conserved. Type IV pili mediate twitching motility, which is important for subsequent spreading of the bacteria over the surface and the formation of microcolonies within a developing biofilm [13]. Twitching motility proteins PilJ and PilK are co-conserved within this cluster and are highly interconnected with flagella and chemotaxis proteins. Flagellar motility appears to be required for approaching surfaces, and 17 flagellar proteins are co-conserved (Figure 4c). Chemotaxis is required for the bacteria to swim towards nutrients associated with a surface. *P. aerginosa *has two chemotaxis signaling systems, and proteins representing both are in the biofilm cluster (CheR1, CheR2, CheA, CheW, PA0173, PA0178; PctA, PctB, PctC). These data suggest that chemotaxis, flagella, and pili proteins may be co-conserved because they all contribute to biofilm formation. Moreover, the inclusion of *P. aerginosa *in the CS-CCC analysis brought pili proteins into the biofilm cluster, suggesting that in some bacteria, all of these processes co-evolved. Thus, CS-CCC can identify co-conserved networks of proteins that function in biochemically distinct pathways but that contribute to complex biological phenomenon.

#### RpoN connects RpoN-regulated proteins with flagella and with type III secretion system proteins

In some of the bacteria studied, RpoN (also known as σ^54 ^or SigL) clustered with RpoN-regulated proteins and flagella proteins are clustered with type III secretion system proteins (Figure 4c). Flagellar proteins are cluster co-conserved with specific components of type III secretion systems (T3SS), which are important for virulence in *Salmonella enterica *serotype Typhimurium LT2, *E. coli *O157, *Shigella flexneri *and *P. aerginosa *[16] (Table 1). The T3SS of *Shigella *is not chromosomally encoded and so was not included in our analysis. The three subunits of the T3SS and flagella that are co-conserved are integral inner membrane proteins of the flagellar or T3SS export apparatus that forms the channel through which proteins are secreted [17]. *S. typhimurium *LT2 and *E. coli *O157 both encode two T3SSes, and the corresponding proteins from each are within this cluster. In *E. coli *K12, *S*. *typhimurium *LT2, and *B. subtilis*, RpoN connects the RpoN-regulated and the flagellar/T3SS clusters. This is consistent with experimental data that flagellar genes (*flhA *and *flhB*) are activated by RpoN [18]. Thus, RpoN likely connects two distinct clusters because it regulates proteins in both clusters. This demonstrates that because CS-CCC examines multiple genomes simultaneously, it has the power to show that proteins unique to particular organisms may function with proteins common to multiple organisms, enabling the placement of unstudied proteins within a broader biological context.

**Table 1:** Homology between co-conserved flagellar and T3SS genes

Flagellar	T3SS
*S. typhimurium *LT2	
*flhA*	*invA; ssaV*
*flhB*	spaS*; ssaU
*fliP*	*spaP; ssaR*
	
*E. coli *0157	
*flhA*	*Z4195, escV*
*flhB*	*Z4185, escU*
*fliP*	*Z4189, escR*
	
*P. aerginosa *(PAO1)	
*fliP*	*pscR*
*flhA*	*pscD*
*flhB*	*pscU*

*spaS in not co-conserved with high cofidence (0.41); the confidence level for the remaining proteins is ≥0.6.

### CS-CCC can be used to assign function to unstudied proteins

#### Genes that function in biofilm formation

**Figure 5:**
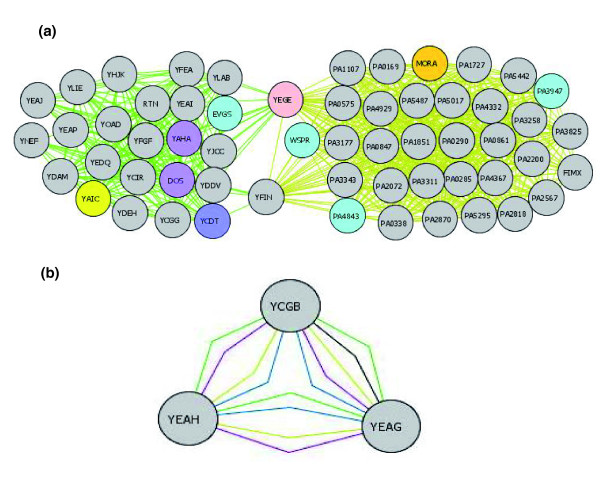
Using CS-CCC to assign protein function. **(a) **Co-conservation of GGDEF and EAL domains across *E. coli *K12 (green edge) and *P. aeruginosa *(mustard edge). Proteins are color coded based on function: motility regulators, orange; sensors, red; RNase II modulators, yellow; two-component response regulators, light blue; diguanylate cyclases, blue; phosphodiesterases, purple; uncategorized, gray. **(b) **Co-conservation of triplet YcgB, YeaH, and YeaG across several species. Edge color code: *E. coli *K12, green; *E. coli *O157, blue; *Shigella flexneri*, black; *S. typhimurium *LT2, purple; *P. aeruginosa*, mustard.

Figure 5a shows two large clusters of proteins built around YegE or YfiN in *E. coli *K12 and *P. aeruginosa*. These clusters are co-conserved with variable numbers of proteins among all of our Gram-negative bacteria. Even though most of these proteins have unknown function, many have GGDEF (Gly-Gly-Asp-Glu-Phe) or EAL (Glu-Ala-Leu) domains, which have been implicated in expression of biofilm phenotypes [19]. Interestingly, each protein of known function within this cluster in PAO1 (WspR, MorA, and FimX) has also been implicated in biofilm phenotypes. WspR is a response regulator that activates pili adhesion genes required for biofilm formation [20]. MorA is a membrane-localized negative regulator of the timing of flagellar formation and plays a role in the establishment of biofilms [21]. FimX is required for a type of twitching motility critical to biofilm formation [22]. FimX is a signal sensing protein with phosphotransfer activity and a GGDEF domain. GGDEF encodes a dinucleotide cyclase that generates cyclic di-GMP and is present in all proteins known to be involved in the regulation of cellulose synthesis. Cyclic di-GMP is a novel bacterial second messenger that directs the transition from sessility to motility [19]. Cyclic di-GMP is degraded by proteins with EAL domains, which are cyclic dinuclotide phosphodiesterases [19]. Proteins containing the GGDEF and EAL domain can regulate biofilm formation and/or cell aggregation by controlling the levels of cyclic di-GMP [19]. Interestingly, most of the proteins in these large clusters have GGDEF or EAL domains. Of the 44 known *P. aeruginosa *proteins with GGDEF or EAL domains [19], 34 are in this cluster; 19 have GGDEF and 15 have EAL domains. *E. coli *K12 has a similar cluster of GGDEF and EAL domains (Figure 5a). The 25 proteins within this cluster are highly interconnected. Of the 38 *E. coli *K12 known GGDEF or EAL domain containing proteins [23], 24 are co-conserved within this cluster. EvgS is a sensor protein for a two component regulatory system [24] that is also within this cluster. Evgs is involved in quorum sensing and may be important in biofilm establishment or maintenance. Over-expression of *evgS *causes abnormal biofilm architecture [25] and previous studies also noted that quorum sensing is involved in biofilm formation [26]. Our experimental data show that four of the GGDEF domain containing proteins in the network of Figure 5a that previously had no known function do indeed mediate biofilm formation [27]. Similar biofilm clusters were identified by the CS-CCC method in all of the Gram-negative bacteria we examined. Thus, by clustering together unstudied proteins, whether or not they have sequence homology, CS-CCC suggests that these proteins may function in a common phenomenon.

#### Small clusters can contain proteins that function in the same processes

Examination of small protein clusters revealed that most pairs or triplets contain proteins that function in the same processes. To further test this observation, we experimentally examined the triplet containing YcgB, YeaH, and YeaG, which cluster together across different bacteria (Figure 5b). Because independent data indicate that *yeaH*, but not *yeaG*, contributes to antimicrobial peptide resistance in *S. typhimurium *[28], we determined whether strains lacking *ycgB *have a similar phenotype. Strains lacking *ycgB *were indeed sensitive to antimicrobial peptides (unpublished data). Thus, CS-CCC analyses revealed previously unknown protein interactions that provided sufficient justification to test a specific biological hypothesis suggested by these interactions.

### When proteins are not identified as co-conserved using CS-CCC

In this study, we have shown that CS-CCC of proteins provides important information. Both the presence and the absence of clustered co-conservation for any given protein are informative. There are at least two reasons why proteins that function together are not co-conserved in a species: first, a protein is found only in certain organisms or a protein function is performed by different proteins in different organisms; and second, a result is a false negative.

#### A protein is found only in certain organisms: T3SS effectors

Effector proteins are secreted by T3SS machinery and function to alter host cell physiology [29]. A bacterial species can have many effectors but they generally do share apparent sequence homology, either within or between bacteria [30]. We examined 49 known SPI2 and SPI1 effectors in *S*. *typhimurium *LT2 and 40 known effectors in *P. aeruginosa *and found that none of these proteins are co-conserved. In contrast, some of the known translocon T3SS proteins, which form the secretion apparatus, are highly co-conserved (Figure 4c). Thus, while CS-CCC offers insights into the function of proteins that are co-conserved, our results show that some of the non co-conserved proteins, such as effectors, are organism specific.

#### A result is a false negative: flagella and RpoN

Our analysis of false negatives reveals that the CS-CCC method produces some false negatives. For instance, there is no co-conservation between RpoN and flagella in *E. coli 0157*, *S. flexneri *and *P. aeruginosa *(Figure 4c). However, it has been experimentally shown in *P. aeruginosa *that many flagellar genes, such as *flhA *and *flhB*, are regulated by RpoN [18]. In addition, an RpoN consensus sequence is located in the intergenic region between *flhB *and *flhA *[23]. These data suggest that the absence of co-clustering of RpoN with flagellar proteins in *P. aeruginosa *is a false negative result. Thus, when proteins are not co-conserved, it cannot be concluded that they are functionally unrelated. This result further underlines the value of developing and comparing interaction networks from multiple genomes when attempting to infer function.

There are also some situations in which a result is both a false negative and the protein in question is found only in certain organisms. The bacterial flagellum is a complex molecular system with multiple components required for functional motility. It extends from the cytoplasm to the cell exterior. Not only are flagella organelles of locomotion, but they also play important roles in attachment and biofilm formation. There are common themes in flagellar protein control and assembly, but there also appears to be variation among organisms. Some of the flagellar proteins are not co-conserved in any of the bacteria of our study, such as, three ring proteins (FlgH, FlgI, and FliF), and some of the axle-like proteins FliE, FlgB, FlgF, FlgL, and FliD. FliE has been shown to physically interact with FlgB [31]. The stator motor proteins MotA and MotB are also not co-conserved. Thus, CS-CCC analysis of the flagellar cluster yields both false negative results and is also a consequence of species-specific proteins. This also illustrates that determining why proteins are not co-conserved can be difficult, without additional information.

## Discussion

Large volumes of data make computational methods feasible, exciting, and preferable to gene-by-gene homology searches. We have shown that use of CS-CCC expands protein interaction networks to include proteins with distinct functions that are involved in coherent biological processes, offers insight into the function of uncharacterized proteins, reveals unique information about each genome examined, and gives insight into the process of evolution.

Protein co-conservation can be a result of many factors, including vertical inheritance or functional selection. Thus, we have examined patterns of CCC within and across several bacteria using CS-CCC. Our analysis showed that this computational approach provides us with more information than the traditional homology approaches or CCC. Homology approaches to protein function are based on similarity to other proteins with known functions and are limited by the fact that many proteins have unknown functions. While homology-based methods can be effective for predicting the functions of remote homologs, these methods perform poorly as the evolutionary distance between homologous proteins increases. Even a sophisticated homology-based method fails to successfully assign functions to most of the proteins for a particular organism. CCC, on the other hand, is not strictly based on homology but is limited by its ability to analyze only a single species at a time. In contrast, CS-CCC examines each cluster across multiple species and reveals interactions that both homology-based methods and CCC fail to identify. Use of CS-CCC allows researchers to extend the protein interaction network to better understand pathways involving multiple proteins with multiple functions. Therefore, the CS-CCC method is a significant advance and will be useful for researches in many different fields of biology.

Prediction by CS-CCC provided us with global views of six complete bacterial genomes. Identification by CS-CCC of proteins that cluster together enabled more accurate predictions of the biological roles that proteins with previously unstudied functions may play. For instance, proteins that function in distinct but coordinated processes can be co-conserved across species even though not all processes occur in all bacteria (Figure 4c). In addition, in large, highly interconnected clusters in which most of the proteins have unknown functions, it is likely that they all function together in a common phenomenon. The GGDEF/EAL cluster is an example of this, as many of the previously unknown proteins in this cluster play roles in biofilm formation (Figure 5a). Even small protein clusters identified by CS-CCC are likely to consist of proteins that function in the same process, as shown by COG/TIGR analysis and experimentally (Figure 5b). These analyses provide evidence that the CS-CCC method is a reliable predictor of functional relationships.

For any given method, there are advantages and disadvantages. The number of false positives and false negatives is a key measurement of accuracy. In our case, the number of false negatives is not possible to estimate without performing many additional laboratory experiments. However, our evaluation of CS-CCC showed that the number of false positives was low. Since this method was evaluated based on our selected bacteria, there may be some bias toward overestimation of accuracy when applied to other organisms, and this remains to be tested. In addition, we have shown that our results can be sensitive to the number of bacteria included in our analysis. Finally, there may be some aspects of the bacteria we chose that are not representative of other bacteria, further reducing the generality of these results. Thus, while the report here represents a compelling demonstration of the value of performing CCC across multiple species, future efforts should be focused on developing better understanding of which and how many organisms to include in CS-CCC studies.

## Materials and methods

### Bacteria used to create CS-CCC graphs

We chose to focus on the Gamma subgroup of proteobacteria because members of this subgroup are among the best characterized, including whole genome sequences and curated datasets of protein functions and interactions. The genomes of five closely related Gamma Gram-negative and one low G+C bacteria (*B. subtilis*) were used to evaluate the CCC method. Substantial experimental data exist for all six bacteria. The gammaproteobacteria included *E. coli *(K12 and O157-O157:H7 EDL933), *S. flexneri *(2a str. 2457T), *S. typhimurium *(LT2), and *P. aeruginosa *(PAO1). *E. coli *(K12) is the most intensively studied Gram-negative bacteria and is the closest studied relative of *P. aeruginosa*, and *S. typhimurimum *LT2. *E. coli *(O157-O157:H7 EDL933) is a clinical isolate from raw hamburger meat implicated in hemorrhagic colitis outbreak, and *S. typhimurium *LT2 causes enteritis in humans. *P. aeruginosa *is an opportunistic pathogen and is the major cause of morbidity and mortality in patients with cystic fibrosis; *P. aeruginosa *PAO1 was isolated from a wound [32]. *P. aeruginosa *is a versatile Gram-negative bacterium that also thrives in soil, marshes and coastal marine habitats, and on plant tissues [32]. *E. coli *K12 diverged 4.5 million years ago (MYA) from O157, an estimated 100 MYA from *Salmonella*, 200 MYA from *Pseudomonas*, and 1,200 MYA from *Bacillus*. Thus, we examined a combination of pathogenic and non-pathogenic organisms that range from closely to distantly related.

### Construction of CS-CCC graphs

**Figure 6:**
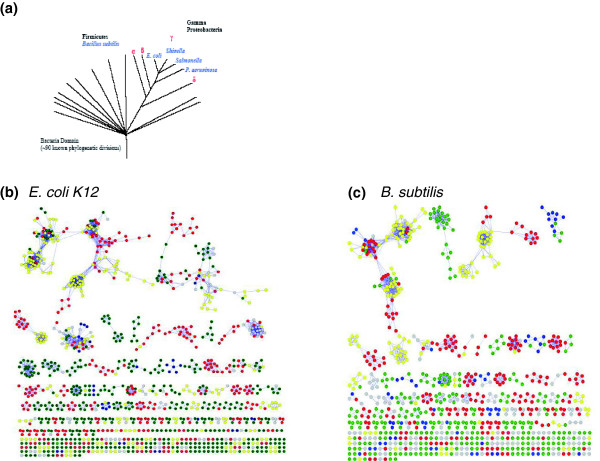
Complete protein-protein interaction network for two organisms. **(a) **Taxonomy of the organisms examined in this study. **(b,c) **Examples of complete protein interaction networks for two of the organisms evaluated here. These figures enable the examination of the size distribution of protein-protein interaction networks in different species. Moreover, proteins are color-coded based on function, thus allowing for the examination of relationships between function and cluster size. For example, this figure shows small or medium size clusters usually contains proteins with similar function. CS-CCC compares all of such networks across multiple species to identify conserved and unique sub-networks. The lengths of the lines in the network hold no meaning and vary simply to facilitate viewing. Cell envelope and cellular process, red; intermediary metabolism, green; information pathway or central dogma, yellow; uncategorized, gray; other, blue.

We began construction of CS-CCC graphs (Figure 1) using predictions of pairwise protein-protein interactions based on phylogenetic profiles (CC methods; Figure 1a). Currently, several databases that compile predictions are available, including Prolinks [6], String [8], and Predictome [5]. We used the Prolinks Database 2.0, which contains a total of 168 microbial genomes, including 10 eukaryotes, 16 Archaea, and 142 Bacteria [6]. Even though ProLinks provides predicted interactions based on a number of different methods (that is, Rosetta stone, gene neighbors, and so on), we have used only interactions prediction by the phylogenetic profiling method in this study. We chose not to use the STRING database as a source of predictions because it conflates co-conservation with orthology information from the COG database [8]; we used COG functional category and TIGR functional role category data to evaluate purely co-conservation inferences. Predictome [5] was not used because it does not provide statistical measures to evaluate the accuracy of each prediction. For each pair assignment (CC), we required a confidence scheme using phylogenetic profiling of at least 60% according to the Prolinks scoring scheme [6]. An E-value of less than 10^-10 ^was used as the threshold for BLASTP in Prolinks to define a homolog of a query protein to be present in a secondary genome. For each bacterial genome analyzed, the number of assigned pairs is shown in Table 2. For each bacterial species, we mapped accession IDs from Prolinks predicted protein pairs to NCBI [33] and then to EcoCyc [23] for *E. coli *K12, *P. aeruginosa *[34] for *P. aeruginosa *and *B. subtilis *[35] for *B. subtilis*. We matched corresponding proteins between species by protein name or synonym. We then constructed CCC graphs using the pairwise links for each species (Figures 1b and 6) using a binary adjacency matrix where 1 indicates the corresponding pair was co-conserved, and 0 otherwise. Networks were represented by graphs in which each node represents a protein and each edge represents an interaction that links two proteins. Network graphs were visualized using Cytoscape [36], an open-source, platform-independent environment software. The lengths of the lines connecting proteins hold no meaning and vary to facilitate viewing of the network. Each network is color-coded based on protein function categories, as described in the corresponding figure legends. The assignment of putative functions was based on EcoCyc, Pseudomonas.com, NCBI and SubtiList, as given in the links above. For separation of connected components of the network and building clusters of proteins, we used breadth-first search (BFS) graph algorithms.

**Table 2:** Comparison of genomes examined in this study

Species name	Genome size	No. of annotated genes	No. (%) of co-conserved genes	No. of co-conserved protein pairs
*E. coli *(K12)	4,639,675	4,242	1,156 (27%)	2,926
*E. coli *(O157-O157:H7 EDL933)	5,528,445	5,324	1,174 (22%)	3,216
*Shigella flexneri *2a str. 2457T	4,599,354	4,068	977 (24%)	4,490
*Salmonella typhimurium *LT2 + pSLT plasmid	4,857,432 + 93,939	4,425 + 102	1,103 (24%)	2,751
*P. aeruginosa *(PAO1)	6,264,403	5,567	1,428 (26%)	5,794
*Bacillus subtilis*	4,214,630	4,105	869 (21%)	1,972

Finally, for comparison of each cluster across different species (CS-CCC), we used BFS to build a network (source network) for a set of target proteins from the source genome. We then built networks for each additional organism that contained proteins with the same name as at least one of the proteins from the source networks. This process identifies proteins and protein interactions that are consistently identified across multiple species (colored gray in Figure 1c) or that are unique to individual species (colored red in Figure 1c). This same method can be used to further parse such networks to identify combined, common and unique networks present for specific proteins across a collection of organisms (Figure 1c). In this way, CS-CCC builds on information generated by CCC (Figure 1b) to provide more accurate and genome-specific protein function assignment. We used protein name to map links across conserved species (thus, links are not explicitly based on orthology) [37-39]. Like all methods, the use of protein names has both advantages and disadvantages. Here, protein name was chosen in order to validate that CS-CCC provides new and biologically informative data not accessible by CCC alone. For this purpose, we chose to validate this method using named proteins where functional information was available. While this is appropriate for method validation, the disadvantage is that there are problems with annotation due in part to a lack of standardization, which would limit the number of proteins for which this analysis can be reliably performed. In light of this limitation, we considered using reciprocal homology as an alternative to protein name. We found that this introduces unacceptable levels of cross-talk, much of which is likely noise. Addressing this limitation is an important area for continued effort.

### Data availability

Data are available upon request.

## Abbreviations

BFS, breadth-first search; CC, co-conservation; CCC, cluster co-conservation; COG, Clusters of Orthologous Groups; CS-CCC, cross-species clustered co-conservation; MCP, methyl-accepting chemotaxis protein; MYA, million years ago; TIGR, The Institute for Genomic Research; T3SS, type III secretion systems.

## Authors' contributions

AK implemented the methods and analyzed the data. CSD interpreted the results. The manuscript was written by AK, CSD and edited by RTG and LH. KDE performed experiments. RTG oversaw all biological aspects of the work and LH supervised the computational aspect.

## Additional data files

The following additional data are available with the online version of this paper. Additional data file 1 is a figure that shows the reliability of predicted protein interaction pairs using TIGR role categories at three different confidence levels.

## Supplementary Material

Additional data file 1 Comparison of TIGR functional categories of predicted pairs at three different confidence levels. The first method (1) used only *E. coli *K12. Each subsequent method added an additional (underlined) bacterial strain. 1, *E. coli *K12; 2, *E. coli *K12 and *E. coli *O157; 3, *E. coli *K12, *E. coli *O157 and *S. flexneri*; 4, *E. coli *K12, *E. coli *O157, *S. flexneri*, and *S. typhimurium *LT2; 5, *E. coli *K12, *E. coli *O157, *S. flexneri*, *S. typhimurium *LT2, and *P. aeruginosa*; 6, *E. coli *K12, *E. coli *O157, *S. flexneri*, *S. typhimurium *LT2, *P. aeruginosa*, and *B. subtilis*. Same functional category (blue); different functional category (green); at least one protein is unclassified (yellow). Click here for file
