# Supplementary figures and images for: Cross-species cluster co-conservation: a new method for generating protein interaction networks
Source: Genome Biol. 2007 Sep 5;8(9):R185. doi: 10.1186/gb-2007-8-9-r185 (PMC2375023; doi:10.1186/gb-2007-8-9-r185)

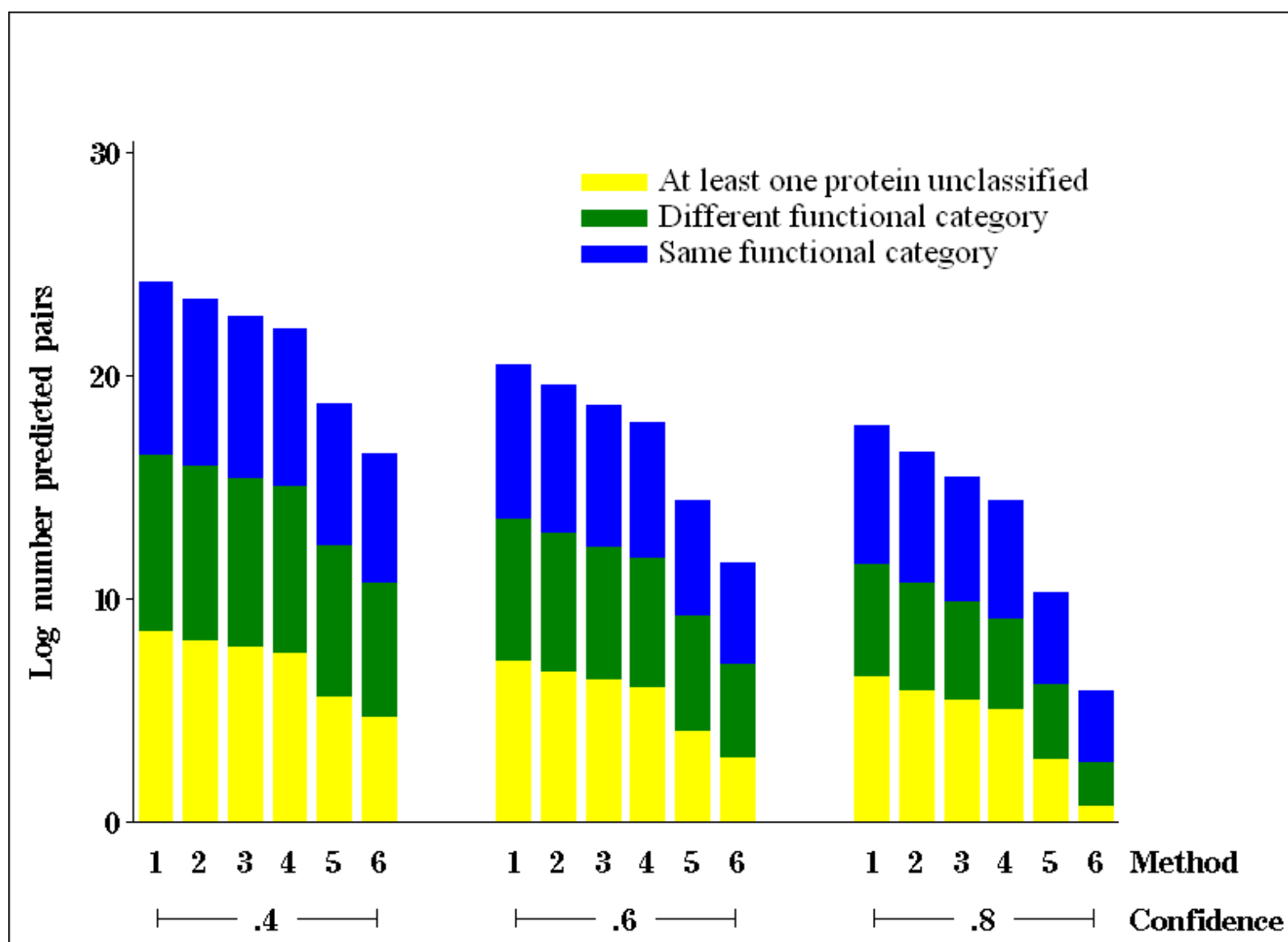

Supplementary Figure 1.

Supplement: Additional data file 1 — Comparison of TIGR functional categories of predicted pairs at three different confidence levels. The first method (1) used only E. coli K12. Each subsequent method added an additional (underlined) bacterial strain. 1, E. coli K12; 2, E. coli K12 and E. coli O157; 3, E. coli K12, E. coli O157 and S. flexneri; 4, E. coli K12, E. coli O157, S. flexneri, and S. typhimurium LT2; 5, E. coli K12, E. coli O157, S. flexneri, S. typhimurium LT2, and P. aeruginosa; 6, E. coli K12, E. coli O157, S. flexneri, S. typhimurium LT2, P. aeruginosa, and B. subtilis. Same functional category (blue); different functional category (green); at least one protein is unclassified (yellow). [file gb-2007-8-9-r185-S1.pdf]
